# Species-specific Microorganisms in Acid-tolerant *Chironomus* Larvae Reared in a Neutral pH Range under Laboratory Conditions: Single Dataset Analysis

**DOI:** 10.1264/jsme2.ME23029

**Published:** 2023-11-08

**Authors:** Sotaro Fujii, Koichiro Kawai, Yoshihiro Sambongi, Satoshi Wakai

**Affiliations:** 1 Graduate School of Integrated Sciences for Life, Hiroshima University, Kagamiyama, Higashi-Hiroshima, Hiroshima, Japan; 2 Diamond Light Source Ltd., Harwell Science and Innovation Campus, Didcot, UK; 3 Seto Inland Sea Carbon-neutral Research Center, Hiroshima University, Higashi-Hiroshima, Hiroshima, Japan; 4 Institute for Extra-cutting-edge Science and Technology Avant-garde Research (X-star), Japan Agency for Marine-Earth Science and Technology (JAMSTEC), Yokosuka, Kanagawa, Japan

**Keywords:** acid tolerance, amplicon sequencing, *Chironomus*, microbiome, saccharide transport

## Abstract

To obtain a more detailed understanding of organismal acid tolerance, the larval microbiomes of 11 *Chironomus* species collected from acidic or neutral pH areas in Japan and reared at pH 7–8 under laboratory conditions were systematically compared using an amplicon sequencing ana­lysis. Evenness values were lower for the larval microbiomes of acid-tolerant *Chironomus* cf. *riparius*, *Chironomus fusciceps*, and *Chironomus sulfurosus* than for eight acid-sensitive species based on an alpha diversity ana­lysis. The lower evenness observed suggested a biased abundance of microorganisms, which was consistent with the identification of *Chironomus* species-specific microorganisms (such as *Agromyces mediolanus* and *Comamonas odontotermitis* related bacteria) with high abundance in acid-tolerant larvae. The abundance of specific microorganisms was also high in the microbiome of acid-tolerant larvae of *Chironomus acerbiphilus* reared at pH 4, but not in that of acid-sensitive larvae. Based on a PICRUSt2 ana­lysis, genes involved in saccharide transport were less abundant in the microbiome of acid-tolerant larvae than in that of acid-sensitive larvae, indicating nutrient-poor acidic environments. Although these results were obtained from single datasets, acid-tolerant larvae appeared to establish *Chironomus* species-specific interactions with microorganisms independent of saccharides, in contrast to acid-sensitive larvae. The present study is the first step towards understanding organismal acid tolerance.

Chironomids (Diptera: Chironomidae) are among the most ubiquitous insects in aquatic habitats during their immature stages. It is estimated that 15,000 chironomid species are distributed worldwide and 2,000 species have been collected in Japan to date (Pinder, 1986; Armitage *et al.*, 1995; Kawai *et al.*, 2012). Some chironomid larvae survive under extreme conditions, such as hypoxia, desiccation, low and high temperatures, high salinity, high heavy metal concentrations, and acidic pH (Hinton, 1951; Pinder, 1986; Salmons *et al.*, 1987; Lindegaard, 1995; Suemoto *et al.*, 2004; Kaiser *et al.*, 2010; Kawai *et al.*, 2019).

Japan is one of the most volcanic countries worldwide, releasing sulfur compounds that contribute to the formation of acidic areas. Among the 226 genera in Chironomidae, *Chironomus* is characterized by a relatively large body size, measuring 3–12‍ ‍mm at the adult stage (Wiederholm, 1989; Nihon yusurika-kenkyukai, 2010), thereby enabling morphological studies. A few endemic *Chironomus* species have been collected from areas with acidic pH, such as lakes and hot springs near active volcanoes in Japan (Table 1 and Supplementary Fig. S1). For example, the larvae of *Chironomus.* cf. *riparius*, *Chironomus fusciceps*, and *Chironomus sulfurosus* collected from acidic areas exhibit acid tolerance at pH 2.0 (Kawai *et al.*, 2019). The larvae of *Chironomus acerbiphilus* collected from an acidic lake survive at pH 0.8 (Kawai *et al.*, 2019). Although *Chironomus yoshimatsui* and *Chironomus kiiensis* larvae inhabit neutral pH areas, they also survive at pH 4.0, but die at pH 3.0 (Kawai *et al.*, 2019).

The inherent acid tolerance of *Chironomus* larvae is attributed to various mole­cular mechanisms. The acid tolerance of *C.* cf. *riparius* larvae may be due to a buffering capacity facilitated by high levels of hemoglobin and elevated ATPase activity (Kawai *et al.*, 2019). The expression of cuticle protein genes was recently shown to be up-regulated in *C. sulfurosus* larvae acclimated to acidic conditions, which conferred a physical barrier against an external acidic environment (Fujii *et al.*, 2021). Additionally, *C. sulfurosus* larvae display chemotaxis towards acidic areas, indicating a response to protons (Fujii *et al.*, 2021).

Apart from the mole­cular machinery, microbiomes coexisting with *Chironomus* larvae contribute to the ability of these larvae to cope with harsh external environmental stresses. For example, *Chironomus transvaalensis* larvae were found to tolerate toxic heavy metals through the microbiome (Senderovich and Halpern, 2013; Laviad-Shitrit *et al.*, 2021). Recent studies reported that microbiomes in the larvae of *C. riparius* and *Chironomus sancticaroli* contributed to microplastic degradation (Palacio-Cortés *et al.*, 2022; Janakiev *et al.*, 2023). However, comparative ana­lyses of microbiomes in the larvae of different *Chironomus* species and their acid tolerance are lacking.

We previously established methods for rearing the larvae of several *Chironomus* species (Kawai and Konishi, 1986; Kawai and Imabayashi, 2003). In the present study, to elucidate the relationship between the microbiome and organismal acid tolerance, the larvae of 11 *Chironomus* species collected from both acidic and neutral areas in Japan were reared under the same laboratory conditions at pH 7–8 and‍ ‍their microbiomes were compared using amplicon sequencing. Although microbiome diversity was previously shown to be lower in several types of insects reared under laboratory conditions than in those in natural environments (Martinson *et al.*, 2017; Tinker and Ottesen, 2021; Janakiev *et al.*, 2023), we detected a difference between the microorganisms of acid-tolerant and acid-sensitive *Chironomus* larvae reared under the same laboratory conditions.

## Materials and Methods

### Collection and classification of *Chironomus* species

*C.* cf. *riparius*, *C. fusciceps* (Taxonomy ID: 1165751), *C. sulfurosus* (Taxonomy ID: 1165750), and *C. acerbiphilus* (Taxonomy ID: 391761) were collected from acidic areas, namely, Usoriyama Lake, Unzen hot spring, Kirishima hot spring, and Katanuma Lake, respectively, between 2002 and 2011 (Kawai *et al.*, 2019) (Table 1 and Supplementary Fig. S1). *C. yoshimatsui* (Taxonomy ID: 169422), *C. kiiensis* (Taxonomy ID: 84408), *Chironomus nippodorsalis* (Taxonomy ID: 1225360), *Chironomus nipponensis* (Taxonomy ID: 586675), *Chironomus javanus* (Taxonomy ID: 391764), *Chironomus okinawanus* (Taxonomy ID: 2138212), *Chironomus flaviplumus* (Taxonomy ID: 586673), and *Chironomus plumosus* (Taxonomy ID: 33397) were collected from neutral areas in Japan between 2009 and 2011 (Kawai *et al.*, 2019) (Table 1).

Eggs laid by a single adult female collected from the natural environment hatched to become 1^st^ instar larvae, which were then reared at pH 7–8 with a milk-agar mat, as described below, until the adult stage. The resulting adult males, with clear morphological differentiation in contrast to that in adult females or immature stages, have been used for species identification, as previously described (Sasa and Kikuchi, 1995; Nihon Yusurika-kenkyukai, 2010). Larvae from the correctly identified *Chironomus* species were reared at pH 7–8 under laboratory conditions; neutral pH is commonly applied for rearing. Only the first generation of *C. plumosus* larvae was available for unknown reasons, whereas other species were reared for several generations.

### Definition of acid tolerance

The larvae of *C.* cf. *riparius*, *C. fusciceps*, *C. sulfurosus*, and *C. acerbiphilus* were able to survive at pH 2.0 and, thus, these species were defined as “acid-tolerant” throughout the present study (Table 1). In contrast, the larvae of *C. yoshimatsui*, *C. kiiensis*, *C. nippodorsalis*, *C. nipponensis*, *C. javanus*, *C. okinawanus*, *C. flaviplumus*, and *C. plumosus* were unable to survive at pH 2.0 and, thus, were defined as “acid-sensitive” (Table 1).

### Rearing conditions

The acid-tolerant and -sensitive larvae of all *Chironomus* species, except for *C. acerbiphilus*, were reared separately in a container (15‍ ‍cm in diameter and 9‍ ‍cm in height; Supplementary Fig. S2, left) with a milk-agar mat constructed using 1% (w/v) agar and 2% (v/v) milk on the bottom (Table 1), which had been autoclaved at 105°C for 5‍ ‍min in advance. The milk-agar mat was required for rearing under laboratory conditions because *Chironomus* larvae have sediment-dwelling features and build nests. The milk-agar mat was soaked in autoclaved tap water in the container covered with nylon cloth (mesh size: 161‍ ‍μm, N-No. 110S; NBC Meshtec) during rearing in the same laboratory room. After hatching, 3^rd^ and 4^th^ instar larvae were reared at 20–25°C and pH 7–8 for one week for *C. javanus*; two weeks for *C.* cf. *riparius*, *C. fusciceps*, *C. sulfurosus*, *C. yoshimatsui* (Supplementary Fig. S2, right), *C. kiiensis*, *C. nippodorsalis*, *C. okinawanus*, and *C. plumosus*; and three weeks for *C. flaviplumus* and *C. nipponensis*. They were then harvested and frozen at –20°C until used.

Acid-tolerant *C. acerbiphilus* larvae were unable to grow on the milk-agar mat at pH 7–8, in contrast to other larvae; therefore, its 3^rd^ and 4^th^ instar larvae were reared for three weeks at 20–25°C in a sterile container with fine sand recovered from their habitats and supplied with autoclaved tap water and vegetable fish food tablets (one tablet container^–1^ week^–1^; PLECO, Kyorin). Under these conditions, the rearing pH was maintained at approximately 4 because fine sand was acidic. Although *C. acerbiphilus* was defined as “acid-tolerant” in the present study, larval rearing conditions (*e.g.*, medium and pH) differed from those of the other larvae and, thus, *C. acerbiphilus* was not included in the comparative ana­lysis and was only used as a reference species. The species also appeared to eat algae on sand particles; however, this was outside the scope of our ana­lysis.

### DNA extraction

Three frozen *Chironomus* larvae were washed three times with 1‍ ‍mL of sterile water to remove exogenously attached microorganisms, and whole larval bodies were manually homogenized with a tip. Therefore, microorganisms that endogenously attached to whole larval bodies, in which larval organs were not specified, were used in subsequent experiments. Total DNA was extracted from homogenized larvae using ISO PLANT II (NIPPON GENE), according to the manufacturer’s instructions for bacterial DNA isolation. Extracted DNA solutions were stored at –20°C until used.

### PCR, library construction, and 16S rRNA gene sequencing

The V3/V4 region of the 16S rRNA gene was amplified from each DNA sample using Illumina341F and Illumina785R primers, which contained Illumina adapter nucleotide sequences, in accordance with the Illumina 16S Metagenomic Sequencing Library Preparation Guide (https://www.illumina.com). The resulting PCR products were cleaned using AMPure XP beads (Thermo Fisher Scientific), and index sequences for multiple ana­lyses were added with Nextra XT Index Kit v2 set A (Illumina). The indexed PCR products were cleaned again using AMPure XP beads, and DNA concentrations were measured using the Qubit High-Sensitivity Assay (Thermo Fisher Scientific). All indexed samples were mixed in a single tube at a concentration of 4 nM. Loading sample pools were prepared at a final concentration of 4 pM, containing a 2 pM PhiX control library (Illumina). Amplicon sequencing was then performed using an Illumina MiSeq platform and MiSeq v3 reagent (Illumina) to obtain 300 bp paired-end reads according to Illumina’s standard protocol.

### Data processing and ana­lysis

Raw FASTQ files were demultiplexed based on each index sequence using the QIIME2 demux plugin (Caporaso *et al.*, 2010). The resulting demultiplexed sequences were denoised using the dada2 denoise-paired command (Callahan *et al.*, 2016). To calculate UniFrac distances, denoised sequences were processed using the following QIIME2 commands: qiime feature-table summarize, qiime feature-table tabulate-seqs, qiime feature-table summarize, qiime feature-table tablate-seqs, qiime alignment mafft, qiime alignment mask, qiime phylogeny fasttree, and qiime phylogeny midpoint-root (Bokulich *et al.*, 2018).

Based on the resulting amplicon sequence variant (ASV) data, rarefaction, alpha and beta diversity, and taxonomic ana­lyses were performed. A rarefaction ana­lysis was conducted using the qiime diversity alpha-rarefaction command. Alpha and beta diversity ana­lyses were performed using the qiime diversity core-metrics-phylogenetic command at a sampling depth of 46,462, resulting in unweighted and weighted UniFrac distances. Diversity metrics were calculated and plotted using the core-diversity and emperor plugins (Vázquez-Baeza *et al.*, 2013). To evaluate differences between acid-tolerant and -sensitive larval microbiomes, a permutational multivariate ana­lysis of variance (PERMANOVA) was conducted based on weighted UniFrac distances using the qiime diversity beta-group-significance command. In the taxonomic ana­lysis, the representative sequence file was processed with the qiime feature-classifier classify-sklearn plugin using the pretrained SILVA-138 99% database. Bar plot data were then generated using the qiime metadata tabulate and qiime taxa barplot commands. Each processed file was visualized with QIIME2 View (https://view.qiime2.org) and principal coordinate ana­lysis (PCoA) plot data were exported using QIIME tools.

The linear discriminant ana­lysis (LDA) effect size (LEfSe) tool (http://huttenhower.sph.harvard.edu/lefse/) with default settings was used to identify microbial taxa with significant differences in abundance between acid-tolerant and -sensitive *Chironomus* larvae. Phylum, class, order, family, genus, and species levels were examined (Segata *et al.*, 2011).

To predict the functional profile of microorganisms in larvae, PICRUSt2 (Douglas *et al.*, 2020) was employed, and the functional annotation of gene sequences was performed based on the Kyoto Encyclopedia of Genes and Genomes (KEGG) orthology (https://www.kegg.jp/kegg/kegg1.html). Genes with significant differences in abundance between microorganisms from acid-tolerant and -sensitive larvae were further analyzed using Welch’s *t*-test. Differences in abundance were considered to be significant with a false discovery rate cut-off of 0.05.

The sequence data used in the present study were deposited in the DDBJ/EMBL/GenBank databases. The datasets were obtained from NCBI with the accession numbers DRR453741–DRR453744 and DRR453746–DRR453753.

## Results and Discussion

### Rationale for larval rearing conditions and sources of microorganisms

Although the rearing containers, milk-agar mats, and tap water used in the present study were autoclaved and covered with a nylon cloth (Supplementary Fig. S2, left), contamination with aerosol microorganisms occurred during the rearing period. However, microorganisms accompanied by *Chironomus* larvae were placed in the container before possible contamination with aerosol microorganisms through the nylon cloth. These conditions were identical for all the‍ ‍rearing containers placed in the same laboratory room, enabling a comparative ana­lysis of microbiomes from the larvae of *Chironomus* species.

Since the microorganisms present in the milk-agar mat or water of each rearing container were not examined in the present study, it was unclear whether the microorganisms were vertically transmitted to larvae across generations or acquired horizontally from rearing environments. In addition, whole larval bodies were used in microbiome ana­lyses after washing; therefore, we were unable to specify the source organs of the microbial taxa. However, differences were observed in microbiomes and microorganisms between the larvae of acid-tolerant and -sensitive species, as described below, from which data were collected under similarly controlled experimental conditions.

### Alpha diversity of microbiomes

**Fig. 1.**
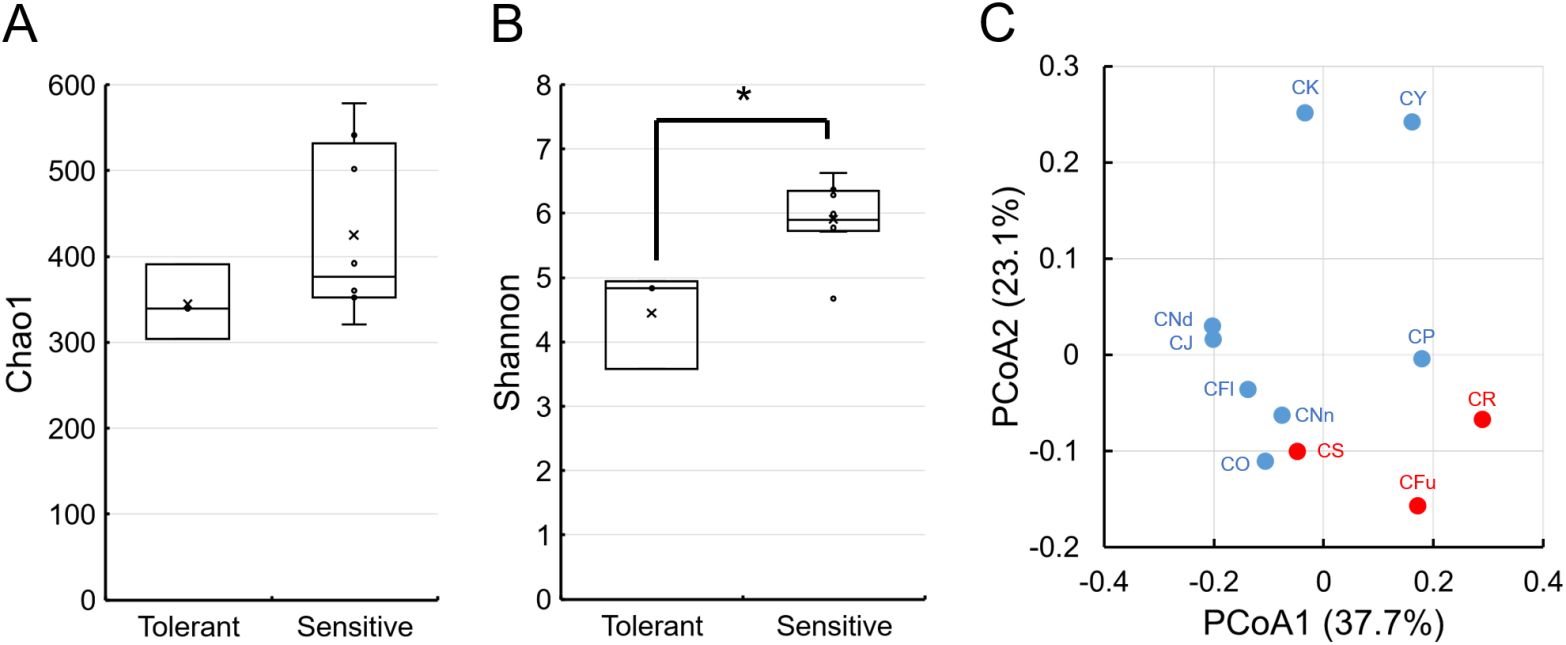
Comparison of microbiomes in acid-tolerant and acid-sensitive larvae of *Chironomus* species. Box-and-whisker plots represent the Chao1 (A) and Shannon (B) indices of acid-tolerant species, excluding *C. acerbiphilus*, (*n*=3) and acid-sensitive species (*n*=8). The central line represents the median, while the top and bottom of the box represent the 25th and 75th percentiles, respectively. Whiskers and cross symbols represent the minimum to maximum values and average, respectively. Welch’s *t*-tests were used to compare results for acid-tolerant and acid-sensitive larvae, and significant differences are indicated with asterisks (*P*<0.03). A principal coordinate ana­lysis (PCoA) plot (C) was based on weighted UniFrac distances and included the following species: *C.* cf. *riparius* (CR), *C. fusciceps* (CFu), *C. sulfurosus* (CS), *C. yoshimatsui* (CY), *C. kiiensis* (CK), *C. nippodorsalis* (CNd), *C. nipponensis* (CNn), *C. javanus* (CJ), *C. okinawanus* (CO), *C. flaviplumus* (CFl), and *C. plumosus* (CP). Red and blue symbols represent acid-tolerant and acid-sensitive larval microbiomes, respectively.

Amplicon sequencing was conducted to gather microbiome data from acid-tolerant and -sensitive *Chironomus* larvae reared at pH 7–8. Following the denoising and removal of chimeric sequences from raw FASTQ data, 972,604 reads were obtained, with 58,071–102,624 reads per sample (Supplementary Table S1). Rarefaction curves were saturated at the subsampling point (58,071) based on alpha and beta diversity ana­lyses (Supplementary Fig. S3). Therefore, these read counts were adequate for a comparative ana­lysis of microbiomes. Chao1 and Shannon indices were computed from amplicon sequencing results for the microbiomes of *Chironomus* larvae. Regarding the acid-tolerant larvae of *C.* cf. *riparius*, *C. fusciceps*, and *C. sulfurosus*, Chao1 and Shannon indices ranged between 306 and 391 and between 3.57 and 4.95, respectively, whereas for the acid-sensitive larvae of *C. kiiensis*, *C. nippodorsalis*, *C. nipponensis*, *C. javanus*, *C. okinawanus*, *C. flaviplumus*, *C. plumosus*, and *C. yoshimatsui*, these indices ranged between 322 and 579 and between 4.68 and 6.63, respectively (Fig. 1A, B and Supplementary Table S1).

**Fig. 2.**
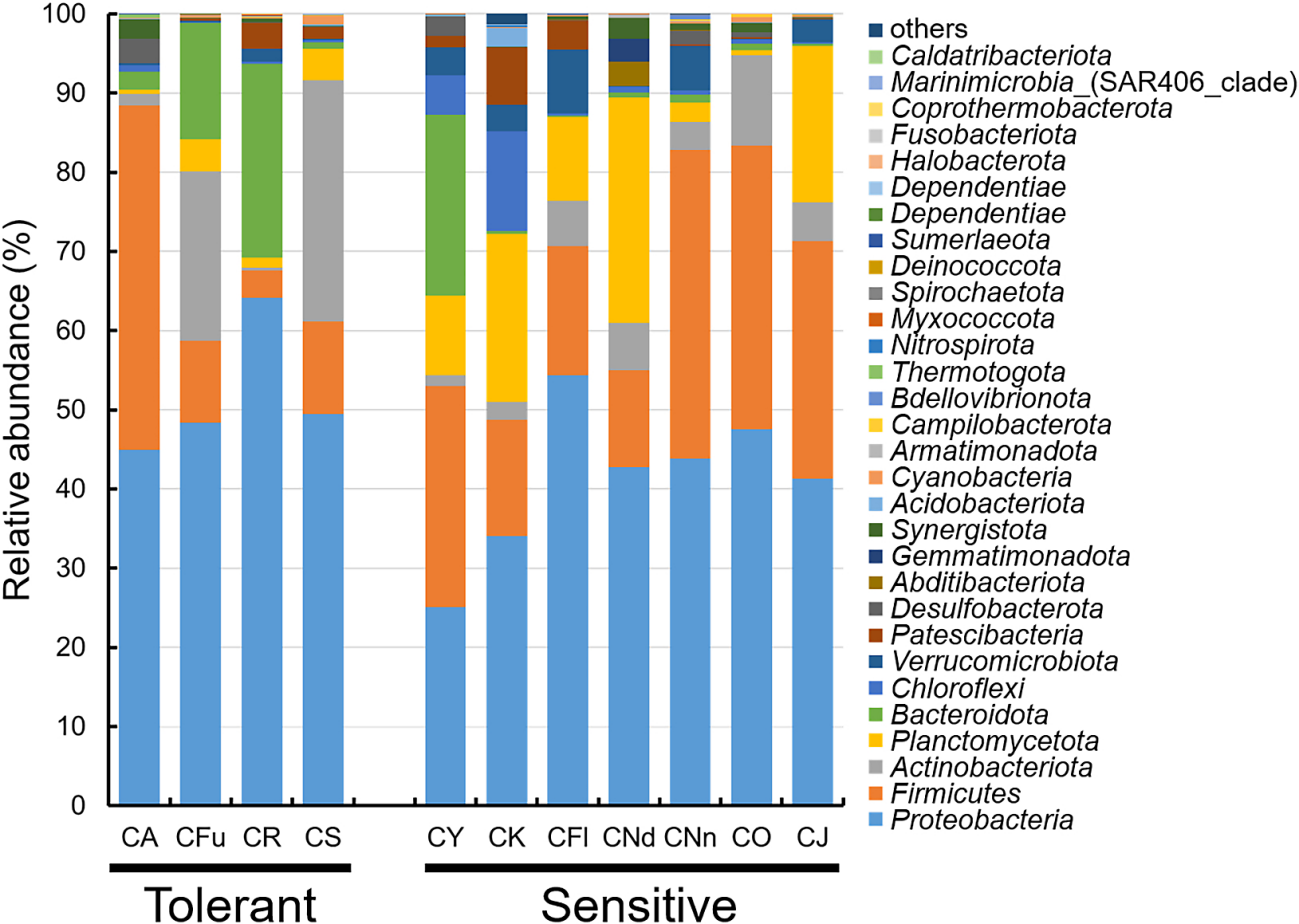
Relative abundances of microorganisms in *Chironomus* larvae at the phylum level. The following species are shown: *C. acerbiphilus* (CA), *C.* cf. *riparius* (CR), *C. fusciceps* (CFu), *C. sulfurosus* (CS), *C. yoshimatsui* (CY), *C. kiiensis* (CK), *C. nippodorsalis* (CNd), *C. nipponensis* (CNn), *C. javanus* (CJ), *C. okinawanus* (CO), *C. flaviplumus* (CFl), and *C. plumosus* (CP).

No significant differences were observed in the Chao1 index between the microbiomes of the three acid-tolerant and eight acid-sensitive *Chironomus* larvae (Fig. 2A and Supplementary Table S1), indicating a similar richness at the ASV level. In contrast, the Shannon index, which represents the evenness of microbiomes at the ASV level, was significantly lower for the microbiomes of acid-tolerant larvae than those of acid-sensitive larvae (Welch’s *t*-test; *P*=0.029, one-sided test) (Fig. 1B and Supplementary Table S1). Lower evenness suggested the lower or higher abundance of various microbial taxa in acid-tolerant *Chironomus* larvae than in acid-sensitive larvae.

### Beta diversity of microbiomes

To further investigate differences among the microbiomes of *Chironomus* larvae, beta diversity was evaluated using PCoA plots based on weighted UniFrac distances for larval microbiomes. Apart from *C. yoshimatsui* and *C. kiiensis*, all points were scattered and did not form clear groups with respect to acid-tolerant and -sensitive *Chironomus* larvae (Fig. 1C). The points for *C. yoshimatsui* and *C. kiiensis* were located far from the others (Fig. 1C). The larvae of these two *Chironomus* species survived under neutral to acidic pH conditions (Kawai *et al.*, 2019); therefore, microorganisms coexisting with the two species were expected to differ from those found in the other species.

### Phylum-level comparisons of microbiomes

Microorganisms were identified to analyze the microbiomes of *Chironomus* larvae. At the phylum level, *Proteobacteria* was found in all microbiomes, with relative abundance ranging from 25.1% in acid-sensitive *C. yoshimatsui* larvae to 64.2% in acid-tolerant *C.* cf. *riparius* larvae (Fig. 2). The relative abundance of *Proteobacteria* did not differ between acid-tolerant and -sensitive larvae. A‍ ‍previous estimate of the relative abundance of *Proteobacteria* in *C. transvaalensis* larvae (27.5%, Senderovich and Halpern, 2013) was within the range of values obtained in the present study (25.1–64.2%). The most prevalent phylum found in the microbiomes of *Chironomus* larvae after *Proteobacteria* was *Firmicutes* (Fig. 2). *Actinobacteriota*, *Planctomycetota*, and *Bacteroidota* were also prevalent in larval microbiomes (Fig. 2). In phylum-level comparisons, acid-tolerant *C.* cf. *riparius* larvae and acid-sensitive *C. plumosus* larvae each had a high relative abundance of *Bacteroidota* (24.5 and 22.9%, respectively), including many anaerobic microorganisms. This result is consistent with the relatively anaerobic habitats of the host *Chironomus* species, such as marshes and eutrophic ponds (Table 1), implying that microorganisms acquired in natural environments were kept under laboratory rearing conditions.

### Specific taxa in microbiomes

**Fig. 3.**
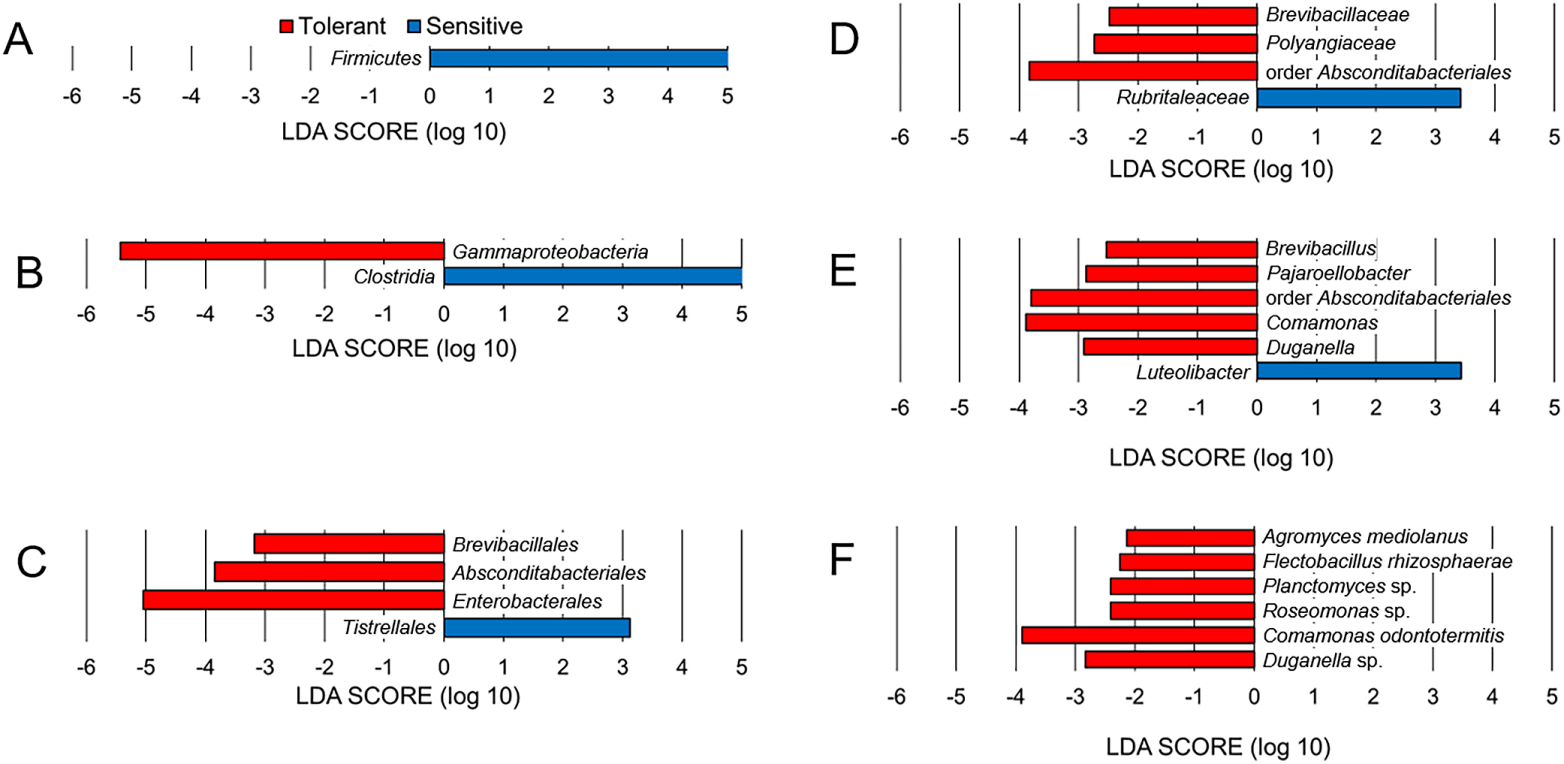
Taxonomic differences between acid-tolerant and sensitive larvae of *Chironomus* species. LDA effect size results at the phylum (A), class (B), order (C), family (D), genus (E), and species (F) levels. Red and blue bars represent the LDA scores (>2.0) for unique taxa in acid-tolerant and sensitive *Chironomus* larval microbiomes, respectively.

The microbiomes of acid-tolerant and -sensitive *Chironomus* larvae were further compared in a statistical framework using LDA LEfSe. At the phylum level, *Firmicutes* was more abundant in acid-sensitive larval microbiomes than in acid-tolerant larval microbiomes (Fig. 3A), whereas *Proteobacteria*, *Actinobacteriota*, and *Bacteroidota* were observed in both acid-tolerant and -sensitive larvae without clear differences in their relative abundance. At the class level, *Gammaproteobacteria* and‍ ‍*Clostridia* were specific taxonomic features in the microbiomes of acid-tolerant and -sensitive *Chironomus* larvae, respectively (Fig. 3B). At the order level, *Brevibacillales*, *Absconditabacteriales*, and *Enterobacterales* were specific taxonomic features in the microbiome of acid-tolerant larvae, while *Tistrellales* was observed in the microbiome of acid-sensitive larvae (Fig. 3C). At the family level, *Brevibacillaceae*, *Polyangiaceae*, and order *Absconditabacteriales* unclassified bacterium were specific taxonomic features in the microbiomes of acid-tolerant larvae, and *Rubritaleaceae* was observed in acid-sensitive larvae (Fig. 3D). At the‍ ‍genus level, *Brevibacillus*, *Pajaroellobacter*, order *Absconditabacteriales* unclassified bacterium, *Comamonas*, and *Duganella* were specific taxonomic features in the microbiomes of acid-tolerant larvae, and *Luteolibacter* was‍ ‍observed in the microbiomes of acid-sensitive larvae (Fig. 3E). At the species level, *Agromyces mediolanus*, *Flectobacillus rhizosphaerae*, *Planctomyces* sp., *Roseomonas* sp., *Comamonas odontotermitis*, and *Duganella* sp. related bacteria were specific taxonomic features in the microbiomes of acid-tolerant larvae (Fig. 3F). These results suggest that acid-tolerant and -sensitive larvae maintained different microorganisms during rearing at pH 7–8 under laboratory conditions.

### Chironomus species-specific microorganisms

**Fig. 4.**
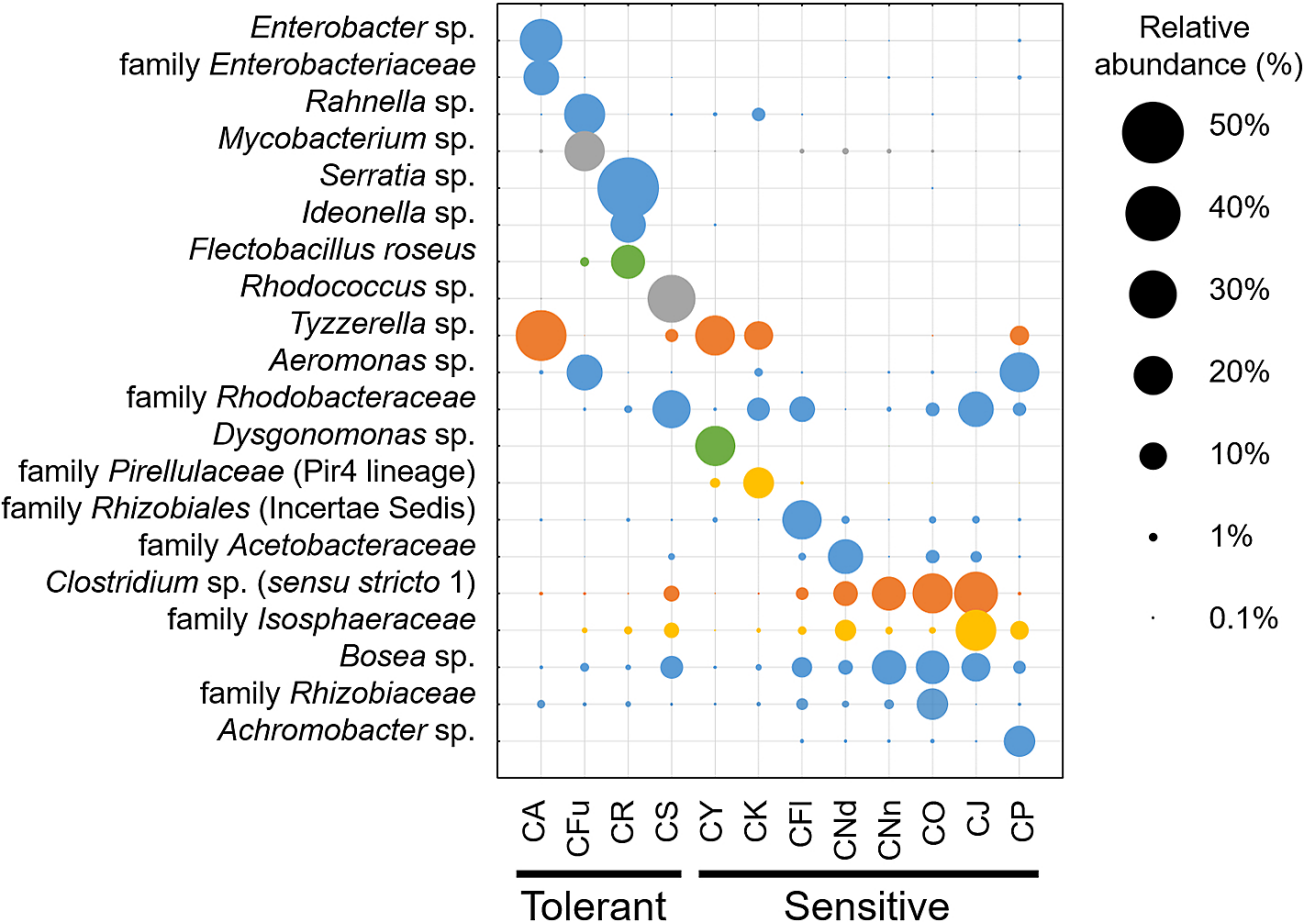
Relative abundances of top twenty representative microorganisms in *Chironomus* larvae at the species level. The following species are shown: *C. acerbiphilus* (CA), *C.* cf. *riparius* (CR), *C. fusciceps* (CFu), *C. sulfurosus* (CS), *C. yoshimatsui* (CY), *C. kiiensis* (CK), *C. nippodorsalis* (CNd), *C. nipponensis* (CNn), *C. javanus* (CJ), *C. okinawanus* (CO), *C. flaviplumus* (CFl), and *C. plumosus* (CP). Balloon colors represent the taxonomic position at the phylum level of each ASV: light blue, *Proteobacteria*; gray, *Actinobacteriota*; green, *Bacteroidota*; and orange, *Firmicutes*. The color scheme used in Fig. 4 is consistent with that shown in Fig. 2 at the phylum level.

We further compared taxa with the top 20 highest relative abundances in the microbiomes of individual *Chironomus* larvae, including *C. acerbiphilus* larvae reared at pH 4 (Fig. 4). Eight microbial species were found almost exclusively in acid-tolerant larvae. These specific microorganisms (with relative abundances) were *Rahnella* sp. (19.6%) and *Mycobacterium* sp. (18.9%) in *C. fusciceps*, *Serratia* sp. (43.7%), *Ideonella* sp. (14.5%), and *Flectobacillus roseus* (13.3%) in *C.* cf. *riparius*, and *Rhodococcus* sp. (26.5%) in *C. sulfurosus*. In the case of *C. acerbiphilus* larvae reared at pH 4, *Enterobacter* sp. (21.1%) and family *Enterobacteriaceae* bacterium (14.6%) were almost exclusively observed. In contrast, the relative abundances of these microorganisms were less than 2.1% in acid-sensitive larvae. Although these results were derived from single datasets without additional statistical support, the microbiomes of acid-tolerant larvae appeared to retain *Chironomus* species-specific abundant microorganisms during laboratory rearing.

Even in acid-tolerant larval microbiomes, acid-tolerant or acidophilic microorganisms were not found. We previously demonstrated that the body fluid pH of acid-tolerant *C. sulfurosus* larvae acclimated at pH 7 and pH 2 was nearly neutral (Fujii *et al.*, 2021). Therefore, acid-tolerant or acidophilic microorganisms may not necessarily exist in acid-tolerant *Chironomus* larvae, and the specific microbiome composition observed in the present study appears to have been affected by factors other than body fluid pH.

### Comparison of functional profiles of microorganisms from acid-tolerant and -sensitive larvae

**Fig. 5.**
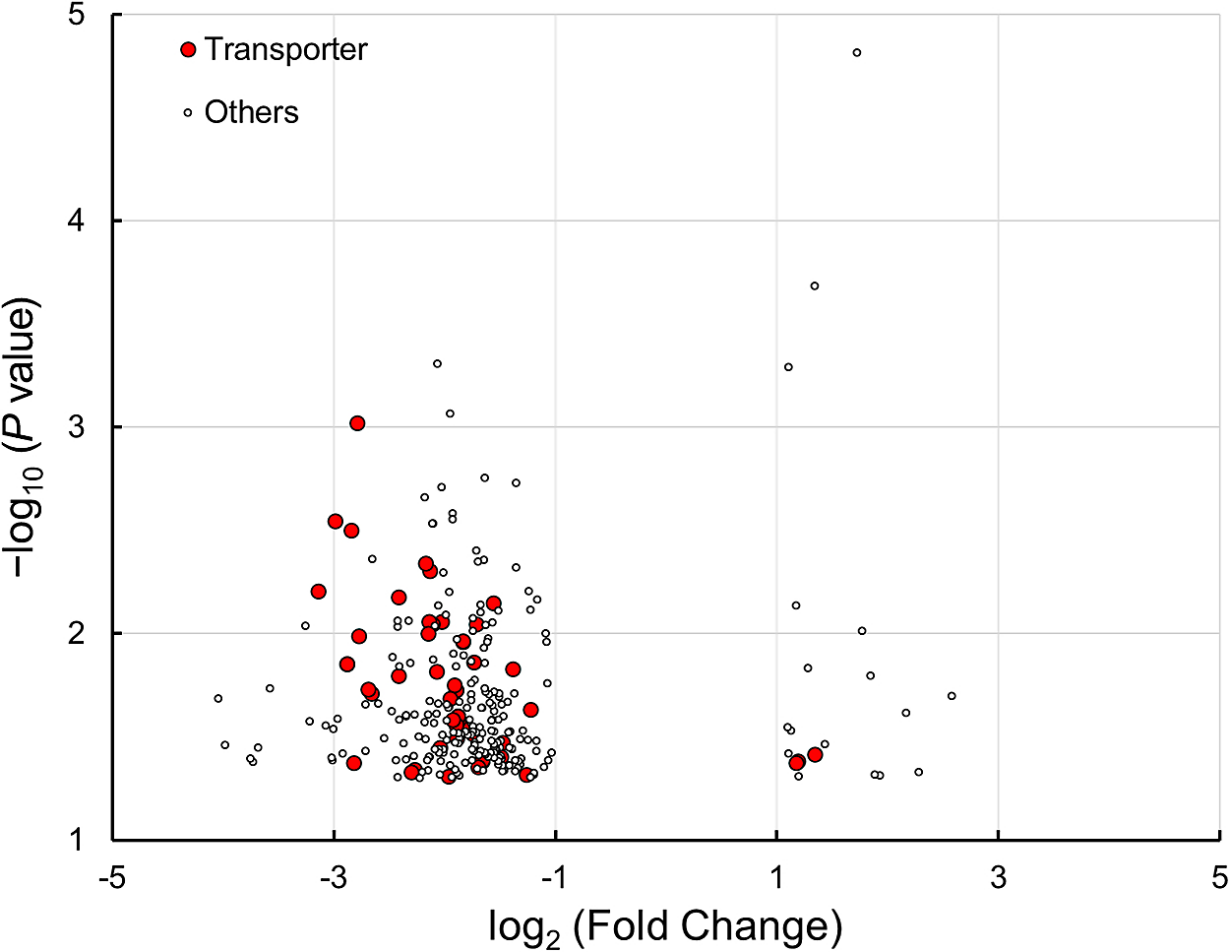
Volcano plot showing genes that were differentially enriched in microbiomes of acid-tolerant and sensitive *Chironomus* larvae. Of the genes detected, 294 genes with significant differences (*P*<0.05, fold change >2) were plotted. Red and white symbols represent transporter and other processes, respectively. Genes with a log_2_ fold change >1 and that <−1 were considered to reflect sufficiently greater and less abundance, respectively, in the microbiomes of acid-tolerant larvae.

A total of 7,391 genes in the microorganisms identified were predicted to be functional using PICRUSt2 and the KEGG database. Among these genes, 294 showed significant differences (*P*<0.05) in abundance between acid-tolerant and -sensitive *Chironomus* larval microbiomes (Fig. 5), with 20 being more abundant in the three acid-tolerant larval microorganisms than in the eight acid-sensitive ones. However, it was difficult to extract a common feature from these 20 genes. In contrast, 51 genes related to transport function were less abundant in the three acid-tolerant larval microorganisms than in the acid-sensitive ones. Among the 51 genes, 20 transporter genes for oligo- and monosaccharides were observed. These results suggested that the microorganisms in acid-tolerant larvae are less dependent on saccharides as energy and carbon sources than those in acid-sensitive larvae. Since organic matter in the sediments where acid-tolerant *Chironomus* larvae inhabit is poorer than that in neutral environments, the microorganisms of acid-tolerant larvae may adapt to host larval habitats. Although the acid-tolerant larvae used herein were reared under neutral conditions, their microorganisms may retain characteristics reflected by the original acidic environments.

To the best of our knowledge, this is the first study to compare the larval microbiomes of acid-tolerant and -sensitive *Chironomus* species. Although our results were derived from single datasets, we obtained several key results: 1) the microbiomes of acid-tolerant *Chironomus* larvae exhibited lower evenness than those of acid-sensitive larvae based on alpha diversity, 2) the microbiomes of acid-tolerant larvae retained *Chironomus* species-specific abundant microorganisms, and 3) the microbiomes of acid-tolerant larvae were less enriched in the genes involved in saccharide transport. Based on these results, we speculate that acid-tolerant *Chironomus* larvae collected from acidic natural environments established species-specific interactions with microorganisms independent of saccharides, in contrast to acid-sensitive larvae, during rearing at pH 7–8 in the laboratory. Comprehensive studies on the holobiome, which comprises microbiomes and acid-tolerant *Chironomus* larvae in acidic environments, will be necessary in the future. This study is the first step towards understanding organismal acid tolerance.

## Citation

Fujii, S., Kawai, K., Sambongi, Y., and Wakai, S. (2023) Species-specific Microorganisms in Acid-tolerant *Chironomus* Larvae Reared in a Neutral pH Range under Laboratory Conditions: Single Dataset Analysis. *Microbes Environ ***38**: ME23029.

https://doi.org/10.1264/jsme2.ME23029

## Supplementary Material

Supplementary Material

## Figures and Tables

**Table 1.** *Chironomus* species used in the present study

Species	Endemic to Japan	Habitat	pH of the collection site	Larvae in laboratory		Reference
				Survival at pH 2.0^a^	Rearing media	
Acid-tolerant						
*C.* cf. *riparius*	No	Marsh, lakes	3.9 (Usoriyama Lake: 41.324N, 141.097E)	Yes	Milk-agar mat	Nihon yusurika-kenkyukai, 2010
*C. fusciceps*	Yes	Acidic streams	2.7 (Unzen hot spring: 32.743N, 130.261E)	Yes	Milk-agar mat	Yamamoto, 1990
*C. sulfurosus*	Yes	Acidic streams	3.2 (Kirishima hot spring: 31.897N, 130.835E)	Yes	Milk-agar mat	Yamamoto, 1990
*C. acerbiphilus*	Yes	Acidic lakes	2.1 (Katanuma Lake: (38.735N, 140.726E)	Yes	Fine sand recovered from Katanuma Lake	Tokunaga, 1939
Acid-sensitive						
*C. yoshimatsui*	Yes	Polluted streams and agricultural canals	n.m.	No	Milk-agar mat	Sasa and Kikuchi, 1995
*C. kiiensis*	No	Rice paddies, ponds, and lakes	n.m.	No	Milk-agar mat	Hashimoto *et al.*, 1981; Sasa and Kikuchi, 1995
*C. nippodorsalis*	Yes	Reservoirs and ponds	n.m.	No	Milk-agar mat	Nihon yusurika-kenkyukai, 2010
*C. nipponensis*	No	Mesotrophic ponds and lakes	n.m.	No	Milk-agar mat	Sasa and Kikuchi, 1995
*C. javanus*	No	Rice paddies and reservoirs	n.m.	No	Milk-agar mat	Hashimoto *et al.*, 1981; Sasa and Kikuchi, 1995
*C. okinawanus*	Yes	Polluted streams and ponds	n.m.	No	Milk-agar mat	Sasa and Kikuchi, 1995
*C. flaviplumus*	No	Rice paddies, ponds, and lakes	n.m.	No	Milk-agar mat	Sasa and Kikuchi, 1995
*C. plumosus*	No	Eutrophic ponds and lakes	n.m.	No	Milk-agar mat	Sasa and Kikuchi, 1995

^a^ Kawai *et al.*, 2019. n.m.: pH not measured, but apparently near neutral.
