# Supplementary material for: Species-specific Microorganisms in Acid-tolerant Chironomus Larvae Reared in a Neutral pH Range under Laboratory Conditions: Single Dataset Analysis
Source: Microbes Environ. 2023 Nov 8;38(6):ME23029. doi: 10.1264/jsme2.ME23029 (PMC10728629; doi:10.1264/jsme2.ME23029)
Supplement: Supplementary file 1 — Supplementary Material [file 38_23029_s1.pdf]

Supplementary materials

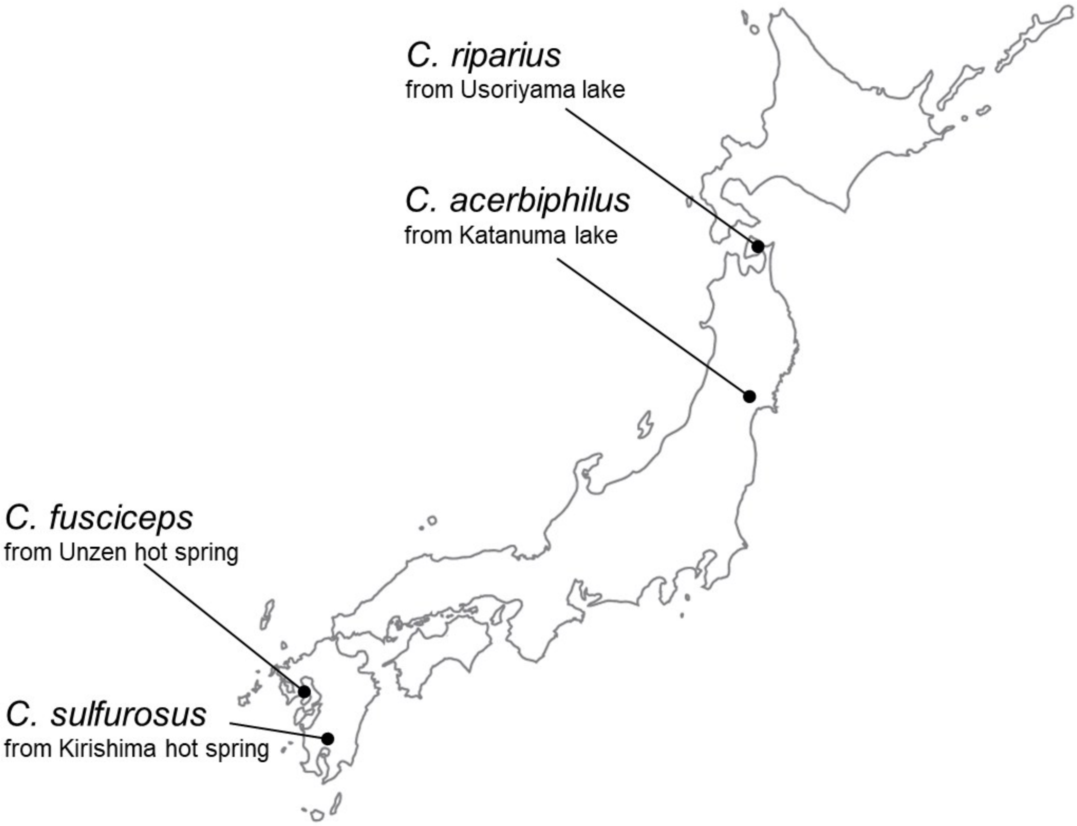

Fig. S1. Collection sites (lakes and hot springs) of four acid-tolerant *Chironomus* species examined in this study.

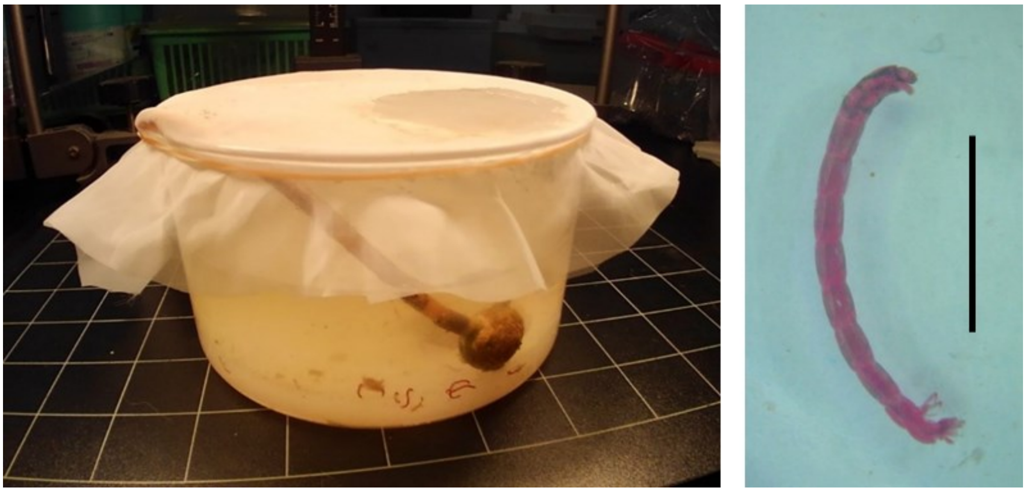

Fig. S2. Rearing container (left) and *C. yoshimatsui* larva (right, scale: 5 mm).

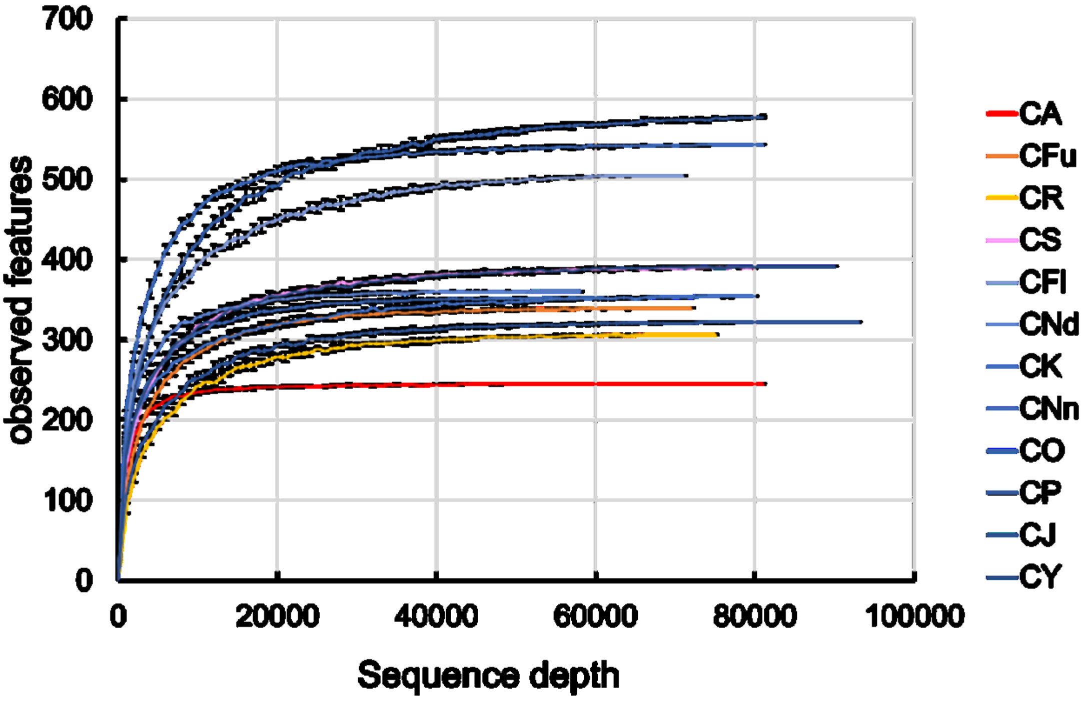

Fig. S3. Rarefaction curves for the *Chironomus* larval microbiomes. *C. acerbiphilus* (CA), *C. fusciceps* (CFu), *C.* cf. *riparius* (CR), *C. sulfurosus* (CS), *C. flaviplumus* (CFl), *C. nippodorsalis* (CNd), *C. kiiensis* (CK), *C. nipponensis* (CNn), *C. okinawanus* (CO), *C. plumosus* (CP), *C. javanus* (CJ), and *C. yoshimatsui* (CY).

Table S1 Sequencing summary

Species	Input (read)	Denoised (read)	Non-chimeric (read)	Chao1 index	Shannon index
Acid-tolerant					
C. acerbiphilus	135,172	87,461	81,304	245	4.64
C. fusciceps	128,432	80,867	72,324	339	4.85
C. cf. *riparius*	139,670	89,981	75,561	306	3.57
C. sulfurosus	179,912	115,959	98,976	391	4.95
Acid-sensitive					
C. yoshimatsui	158,381	97,385	90,784	391	5.80
C. flaviplumus	163,311	100,134	71,777	504	6.36
C. nippodorsalis	137,556	60,104	58,071	360	6.29
C. kiiensis	150,791	90,283	80,709	354	5.99
C. nipponensis	149,472	94,674	85,469	543	6.63
C. okinawanus	119,722	71,496	61,944	352	5.77
C. plumosus	178,040	115,544	102,624	579	5.72
C. javanus	191,832	114,538	93,061	322	4.68
